# Emerging Angiostrongyliasis in Mainland China

**DOI:** 10.3201/eid1401.061529

**Published:** 2008-01

**Authors:** Shan Lv, Yi Zhang, Peter Steinmann, Xiao-Nong Zhou

**Affiliations:** * Chinese Center for Disease Control and Prevention, Shanghai, People’s Republic of China; † Swiss Tropical Institute, Basel, Switzerland

**Keywords:** angiostrongyliasis, *Angiostrongylus cantonensis*, epidemiology, outbreak, China, dispatch

## Abstract

Our review of angiostrongyliasis in China found that the disease is emerging as a result of changes in food consumption habits and long-distance transportation of food. Enhanced understanding of angiostrongyliasis epidemiology, increased public awareness about the risks associated with eating raw food, and enhanced food safety measures are needed.

*Angiostrongylus cantonensis* was first described as a parasite of the Norway rat (*Rattus norvegicus*) and the black rat (*R. rattus*) in Guangzhou (formerly Canton), People’s Republic of China, in 1933. The first human case was reported from Taiwan in 1945. Transmission to humans is primarily by consumption of raw snails. Contaminated vegetables and paratenic hosts such as freshwater prawns, crabs, and frogs may also play a role in transmission (*1*).

The first case of human angiostrongyliasis in mainland China was diagnosed in 1984 (*2*). During the past decade, the number of cases has sharply increased (*3*). A large outbreak occurred in Beijing during 2006. The outbreak peaked during August and involved 160 persons, 100 of whom were hospitalized. This number of patients is similar to the total number of infections recorded in China over the past decade. Our aim was to briefly review angiostrongyliasis outbreaks in mainland China, update angiostrongyliasis epidemiology, and recommend measures to prevent and control angiostrongyliasis.

## The Study

The first reported outbreak of angiostrongyliasis in China occurred in 1997 in the city of Wenzhou in the eastern coastal Zhejiang province; it affected 65 persons (*4*). Since 2000, 6 more outbreaks have been reported, along with numerous individual cases. A literature review found 334 recorded cases; only 4 cases were reported between 1984 and the 1997 outbreak in Wenzhou city. The 7 outbreaks summarized in Table 1 accounted for 86.5% of the total cases.

**Table 1:** Reported angiostrongyliasis outbreaks in mainland China since 1997

Year	Location (city, province)	No. infections	Source of infection	Reference*
1997	Wenzhou, Zhejiang	65	*Pomacea canaliculata†*	Zheng RY et al., 2001
2002	Changle, Fujian	8	*P. canaliculata†*	Lin JX et al., 2003
2002	Fuzhou, Fujian	9	*P. canaliculata†*	Yang FZ et al., 2004
2002	Fuzhou, Fujian	13	*Achatina fulica‡*	Wu CH et al., 2004
2004	Kunming, Yunnan	25	*P. canaliculata†*	Han JH et al., 2005
2005	Kunming, Yunnan	9	*P. canaliculata†*	Wei LP et al., 2005
2006	Beijing, Beijing	160	*P. canaliculata†*	Not available

*Full reference details are available from the corresponding author upon request. †Common name, apple snail. ‡Common name, giant African land snail.

The Figure depicts the 9 provinces in China where angiostrongyliasis has been reported thus far. The youngest patient was 11 months of age, and the oldest was 70 years of age. In the recent Beijing outbreak, 99 of the 100 hospitalized patients were available for interview; 59 were male and 40 were female, age range was 13–57 years, median age was 36 years. The causative agent was confirmed for 16 (4.8%) of 334 cases. Four children died. Outbreak investigations found that 75.1% of all patients had eaten raw apple snails (*Pomacea canaliculata*) or raw giant African land snails (*Achatina fulica*).

**Figure:**
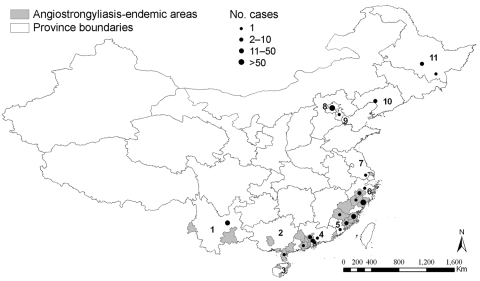
Provinces in People’s Republic of China where cases of angiostrongyliasis has been reported and locations where outbreaks occurred. Province names: 1, Yunnan; 2, Guangxi; 3, Hainan; 4, Guangdong; 5, Fujian; 6, Zhejiang; 7, Jiangsu; 8, Beijing; 9, Tianjing; 10, Liaoning; 11, Heilongjiang.

*A. cantonensis*–endemic foci have been discovered in the provinces of Fujian, Guangxi, Hainan, Yunnan, and Zhejiang (Figure). Jinhua city in Zhejiang province is the parasite-endemic setting furthest north. Infected *A. fulica* were found in a farm (used for temporary cultivation and selling of snails) in Liaoning province in northeast China; however, the infected snails might have been imported from provinces located further south.

Thirty-two species of wild mollusk in China have been screened for *A. cantonensis;* 22 of these species (68.8%) harbored the parasite (Table 2). The highest rate and intensity of infections were recorded in *A. fulica*, followed by slugs (*Vaginulus* spp.) and *P. canaliculata*. Terrestrial snails and slugs showed higher rates and intensities of infections than freshwater mollusks. However, at least 1 freshwater snail, *P. canaliculata,* plays an important role in the epidemiology of angiostrongyliasis.

**Table 2:** Known intermediate hosts of *Angiostrongylus cantonensis* in mainland China

Species	First investigation, y, place	First observation of positive specimens, y, place	Highest recorded prevalence, %, place	Reference*
*Achatina fulica*†‡	1979–1982, Guangzhou	1979–1980, Guangzhou	96.8, Zhongshan	Ding BL et al., 1982 Liang HK et al., 1984 Liang HK et al., 1992 Zhang HM et al., 1996
*Pomacea canaliculata*§¶	1988, Hekou	1997, Cangnan	69.4, Cangnan	Li FH et al., 1989 Pan CW et al., 2002 Li YS et al., 2001
*Camaena cicatricosa‡*	1979–1982, Guangzhou	1979–1982, Guangzhou	50.0, Zhongshan	Ding BL et al., 1982 Liang HK et al., 1984 Liang HK et al., 1992
*Vaginulus* sp.#	1980–1982, Guangzhou	1980–1982, Guangzhou	49.2, Guangzhou	Ding BL et al., 1982
*Phiolomycus bilineatus#*	1979–1982, Guangzhou	1979–1982, Guangzhou	100, Guangzhou	Ding BL et al., 1982 Liang HK et al., 1984
*Deroceras leave#*	2005, Lianjiang/Nan’an	2005, Lianjiang/Nan’an	23.8, Lianjiang/Nan’an	Li LS et al., 2006
*Vaginulus yuxjsjs* sp. nov#	1988, Hekou	1988, Hekou	21.0, Hekou	Li FH et al., 1989
*Ariophantidae* spp.‡	1988, Hekou	1988, Hekou	12.0, Hekou	Li FH et al., 1989
*Macrochlamys loana#*	2005, Lianjiang/Nan’an	2005, Lianjiang/Nan’an	11.2, Lianjiang/Nan’an	Li LS et al., 2006
*Limax flavus#*	2005, Lianjiang/Nan’an	2005, Lianjiang/Nan’an	10.1, Lianjiang/Nan’an	Li LS et al., 2006
*Bradybaena brevispira‡*	1979–1982, Guangzhou	1979–1980, Guangzhou	8.3, Nanhai	Liang HK et al., 1984 Liang HK et al., 1992 Pan CW et al., 2002
*Meghimatium bilinestum‡*	2005, Lianjiang/Nan’an	2005, Lianjiang/Nan’an	5.9, Lianjiang/Nan’an	Li LS et al., 2006
*Trichochloritis rufopila‡*	1986–1990, Zhuhai	1986–1990, Zhuhai	5.8, Zhuhai	Liang HK et al., 1992
*Trichochloritis hungerfordianus‡*	1986–1990, Zhuhai	1986–1991, Zhuhai	5.7, Zhuhai	Liang HK et al., 1992
*Vaginulus alte#*	1988, Hekou	1988, Hekou	4.2, Hekou	Li FH et al., 1989
*Bellamya* spp.¶	2005, Lianjiang/Nan’an	2005, Lianjiang/Nan’an	4.1, Lianjiang/Nan’an	Li LS et al., 2006
*Bellamya aeruginosa*¶	2004, Minhou/Lianjiang	2004, Minhou/Lianjiang	3.8, Minhou	Lin JX et al., 2005
*Bradybaena similaris‡*	1980–1982, Guangzhou	1980–1982, Guangzhou	3.2, Guangzhou	Ding BL et al., 1982
*Bradybaena ravida‡*	2005, Lianjiang/Nan’an	2005, Lianjiang/Nan’an	3.1, Lianjiang/Nan’an	Li LS et al., 2006
*Plectotropis appanata‡*	2005, Lianjiang/Nan’an	2005, Lianjiang/Nan’an	2.6, Lianjiang/Nan’an	Li LS et al., 2006
*Bellamya quadrata*¶	1986–1990, Panyu	1986–1990, Panyu	2.5, Panyu	Liang HK et al., 1992

*Full reference details are available from the corresponding author upon request. †Common name, giant African land snail. ‡Land snail. §Common name, apple snail. ¶Freshwater snail. #Slug.

Of 15 wild rodent species captured in mainland China, 11 harbored *A. cantonensis*. The prevalence and intensity of infection were generally higher in *R. norvegicus* than in other rodents. Infections were also found among nonhuman primate, equine, and canine species (*5**,**6*). However, the prevalence in domestic animals and nonrodent wildlife remains to be fully investigated.

During surveys of 12 potential paratenic host species in China, *A. cantonensis* larvae were recovered from frogs (*Hylarana guentheri*, *Rana limnocharis,* and *R. plancyi*) and toads (*Bufo melanostictus*). In contrast to the situation on different Pacific islands (*7**,**8*), *A. cantonensis* has not yet been found in freshwater shrimp, fish, crabs, and planariae in mainland China.

The *A. cantonensis–*endemic area in China is rapidly expanding, and causes are multifactorial. First, *A. fulica* and *P. canaliculata* have invaded the southern part of China after being imported from East Africa in the 1930s and South America (through Taiwan) in the 1980s, respectively. While both snail species were imported as food, they also became established in the wild fauna and are now common in southern China. Second, vast habitats with suitable environmental conditions can be found in many parts of the country. Recent studies indicate suitable habitats not yet colonized; hence, further expansion of *A. cantonensis–*endemic settings is cause for concern (*9*). Finally, the low host specificity of *A. cantonensis* further supports the expansion of this parasite into new areas.

The predominant freshwater and land snail species found in Chinese markets are *P. canaliculata,* *A. fulica,* *Cipangopaludina chinensis,* and *Bellamya aeruginosa*. The first 2 account for most *A. cantonensis* infections in mainland China. In Taiwan, *C. chinensis* plays an important role in the epidemiology of angiostrongyliasis (*10*). Recently, *B. aeruginosa* was found to harbor natural infections with *A. cantonensis;* several infections have been linked to this species (*11*,*12*). Consumption of freshwater shrimp and crabs has increased over consumption of snails. Although only few *A. cantonensis* infections could be traced to shrimp and crabs and none yet in China, their infection potential merits attention.

## Conclusions

The booming economy and rapid infrastructure development in China have affected food production and trade. For example, these developments enabled the countrywide marketing of aquaculture products and snails produced in the southern provinces, which might also be responsible for the emergence of foodborne trematodiasis (*13*). After the recent angiostrongyliasis outbreak in Beijing, the source of infection was traced to *P. canaliculata* originating from Guangxi province in southwest China. Socioeconomic changes have also increased the popularity of specific consumption habits across China. Although the residents of the southeastern coastal areas have a long history of eating raw food, including snails and seafood, similar dishes have recently become popular among the urban middle and upper classes in inland China.

After the 2006 outbreak in Beijing, different measures have been proposed for preventing and controlling angiostrongyliasis. First, food safety and transportation must be improved to avoid human infections and the further spread of intermediate host snails to areas in which the disease is not endemic. In snail farms, rigorous control of rats should be implemented. Collection and marketing of wild snails should be limited. Second, hygiene and food preparation techniques in restaurants should be improved to prevent cross-contamination of other food items. Third, these efforts should be accompanied by sound information in communication and education campaigns to raise public awareness. The basic message, that consumption of raw or undercooked snails is a key risk factor for the transmission of a serious disease, can be easily conveyed and is readily understood by the public. After the first outbreak in Wenzhou city in 1997, a comprehensive health education campaign about safe food consumption habits and the most prominent symptoms associated with angiostrongyliasis was launched in Zhejiang province. Few cases were reported thereafter.

Awareness of angiostrongyliasis needs to be improved for consumers and health professionals. Education campaigns should inform consumers about the risk of contracting angiostrongyliasis, e.g., by eating raw snails, and professional knowledge among healthcare providers should be improved to ensure timely detection of infections and adequate medical response.
